# Universities and primary care organisations working together to recruit GPs: a qualitative evaluation of the Enfield clinical teaching fellow programme

**DOI:** 10.3399/bjgpopen18X101361

**Published:** 2018-04-24

**Authors:** Melvyn, M Jones, Nadia Bashir, Neetha Purushotham, Rachel Friel, Joe Rosenthal

**Affiliations:** 1 Senior Lecturer in General Practice, Research Department of Primary Care and Population Health, UCL Medical School (Royal Free Campus), London, UK; 2 GP and GP Trainer, Warden Lodge Surgery, Cheshunt, UK; 3 GP, Morecambe Surgery, London, UK; 4 Clinical Teaching Fellow, Research Department of Primary Care and Population Health, UCL Medical School (Royal Free Campus), London, UK; 5 GP, Apollo Health Armadale, Armadale, Australia; 6 Clinical Teaching Fellow, Research Department of Primary Care and Population Health, UCL Medical School (Royal Free Campus), London, UK; 7 GP, Carlton House Surgery, Enfield Town, UK; 8 Clinical Teaching Fellow, Research Department of Primary Care and Population Health, UCL Medical School (Royal Free Campus), London, UK; 9 Department Head of Teaching and Sub-Dean for Community Based Teaching, Research Department of Primary Care and Population Health, UCL Medical School (Royal Free Campus), London, UK; 10 GP, Partnership Primary Care Centre, London, UK

## Abstract

**Background:**

General practice recruitment is in difficulty in the UK as many experienced GPs retire or reduce their commitment. The numbers of junior doctors choosing to specialise in the discipline is also falling, leading to primary care workforce issues particularly in 'hard to serve' areas.

**Aim:**

To evaluate an academic service collaboration on GP recruitment between a primary care organisation (PCO), Enfield CCG, and a university, University College London (UCL).

**Design & setting:**

Evaluation of an academic service collaboration in the Enfield CCG area of north east London.

**Method:**

An action research method utilising qualitative methodology was used to evaluate a local service intervention, undertaken by the participants themselves. The qualitative data were analysed by one researcher but themes were agreed by the whole team. Enfield CCG, an NHS PCO, funded a collaboration with UCL to employ five GPs as clinical teaching fellows to work in Enfield, to increase patients' access, to provide input to CCG development projects, and to provide undergraduate medical student teaching in practice.

**Results:**

Five teaching fellows were employed for ≤2 years and provided 18 266 extra appointments, engaged with development projects, and delivered local undergraduate teaching. The themes identified by stakeholders were the challenges of these organisations working together, recruiting GPs to an underserved area, and perceptions of the model’s value for money.

**Conclusion:**

The evaluation showed that the collaboration of an NHS PCO and a higher education institution can work, and the prestige of being associated with a universty and clinical variety ensured GP recruitment in an area that had previously struggled. However, the project’s costs were high, which affected perceptions of its value.

## How this fits in

Recruitment of GPs to 'hard to serve' areas (such as inner cities and isolated rural communities) is an international problem and impacts on access to care for more vulnerable groups of patients. Using the prestige of a university to help recruit for primary care posts in such areas has been explored in many settings, but these models have often not been sustainable. This study evaluates a model funded by a PCO with commissioning responsibilties, identifies some of the challenges of organisations working together, and explores the issues around sustainability and costs.

## Introduction

Recruitment to UK NHS general practice is an ongoing concern, particularly in inner city or more deprived communities.^1,2^ The London Borough of Enfield is a socially-mixed area comprising both affluent and highly deprived suburbs. The borough historically has struggled to recruit and retain GPs. The most recent data (published in 2015) suggest that Enfield has 0.55 GP whole time equivalents (FTE) per 1000 population, which is below the London average of 0.57, and that there are '245 GP Performers across Enfield of which 25% (*n* = 61) are locums and 23% (*n* = 57) are more than 60 years.'^3^

One of the questions which has arisen in this context is whether there is a potential role for universities' departments of general practice in helping to recruit GPs into 'hard to serve' areas. There are current national initiatives to address this issue in the UK,^4^ and various local models have been piloted, including programmes linking GP posts to universities.^5–9^ A systematic review of GP recruitment identifies several international programmes that combine an academic link with clinical service.^10,11^ There are UK examples; from London in the 1990s, arising from the Tomlinson report and London Implementation Zone (LIZ),^12,13^ which fostered projects such as the Inner City Lecturer scheme,^13^ the London Implementation Zone Educational Initiative,^14,15^ the London Academic Training Scheme (LATS),^16^ and the South London Vocational Trainee Associates scheme.^17^ Similar models have been employed in 'hard to serve' areas, in South Wales^9^ and Durham.^5^ More recently, there have been reports of university-linked localities,^18,19^ and CCGs have been directly employing doctors.^20^

These models have often been used by new GPs as a 'taster post' for a working in a specific community.^5^ However, there is little evidence that these approaches are financially sustainable in the long-term, and there is mixed evidence on long-term GP recruitment.^10^

English primary care structures have undergone substantial reorganisation since the LIZ and LATS programmes of the 1990s.^21^ CCGs now have a statutory role in commissioning secondary care, but many CCGs are increasingly developing a contractor relationship (so-called 'fully delegated responsibility') with primary care.^22^ These changes mean that CCGs and PCOs can now directly influence recruitment in primary care. Will these changes make these academic PCO models more sustainable?

Enfield NHS CCG is a PCO that commissions care. It allocated funding for a 2-year project and sought to collaborate with UCL in appointing four GP clinical teaching fellows (CTFs) to meet several service objectives.

The main aims for this initiative were to improve access by providing 17 000 extra GP appointments over 2 years; to deliver service improvements through research and re-design in priority service development area; and to raise the profile of Enfield for newly qualified GPs to settle in the long term.^23,24^

The purpose of this evaluation is to examine the aims of the project and to assess success against its objectives. No specific funding was allocated to evaluate the project, so one of the implicit objectives was that the CTFs should evaluate the project themselves.

## Method

This is an evaluation of a service innovation.^25,26^ An action research approach was taken,^27^ which is 'a collaborative process between researchers and people in the situation, a process of critical inquiry, a focus on social practice, and a deliberate process of reflective learning'.^28^ Analysis of project documents and reports was used to capture stakeholders’ views about meeting project objectives. A qualitative methodology was utilised, through interviews with key stakeholders.^29,30^ Three of the CTFs acted as the action researchers and recorded interviews. The taped interaction between CTFs themselves and interviewees often generated rich discussion of the project, and so forms part of the data. The recordings were either transcribed verbatim, or annotated and summarised. The interviews were manually analysed using the thematic framework.^30^ One author initially analysed the qualitative data using the following steps: familiarisation, identifying a thematic framework, indexing, charting, mapping, and interpretation. Throughout this process, transcripts were repeatedly re-read to focus on specific points and ideas, to verify the presence of the themes that are identified, and to ensure the context of the themes has been preserved. Themes were then discussed among the team and with key stakeholders. Divergent views were resolved by discussion. Drafts of the manuscript were shared with the CCG GP transformation subgroup to ensure, as the funder and a key stakeholder, that its perspective was captured.

The project is reported in line with COREQ guidelines (further information available from the author on request).^31^

### Recruitment

Participants were identified by the steering group selecting a purposive sample from each disciplinary group. If individuals did not respond to an email, a follow-up reminder was sent 2 weeks later. If they still did not respond, another individual from the same disciplinary group was approached. Participants received a study information leaflet and consent sheet to participate, to audio record, and to use quotes. The main ethical and methodological issue was that the researchers were also participants in the project. A grid was devised that would minimise potential conflicts or collusion through assigning researchers to interview stakeholders with whom they had had minimal role contact during the project. This pragmatic solution is discussed under study limitations.

## Results

Several key documents were identified, including the CCG’s own internal project final report. Other documents included project strategy and interim project management reports.

The CCG funded four full-time GP posts recruited for 2 years and employed by the university. GP CTFs were recruited by national advertisement, and with a joint interview and selection panel. The post attracted a strong field of applicants and four high calibre candidates were appointed. Their experience level ranged from newly qualified to 3 years post-training, and none had previously worked in the Enfield area. One of those appointed took maternity leave early on and did not subsequently return. Her funding was later used to appoint a fifth CTF, but this post was outside the scope of the collaboration. The CTFs’ working week comprised five clinical GP sessions, two sessions for CCG service development, two undergraduate teaching sessions, and one session for professional development. Each CTF rotated to a different practice halfway through the 2-year programme in order to broaden their experience and share the additional workforce benefit that the CTF represented among different practices.

All practices in Enfield were invited to apply to host the CTF posts. Smaller, non-teaching practices were particularly encouraged to apply. Host practices were selected by application and eight were chosen. Service development projects were identified by the CCG and undergraduate teaching was introduced into 'teaching-naive' practices.

The CCG project internal summary report stated that the project delivered two of its main aims (unpublished data), but withheld judgment on third aim.

For the qualitative element of the evaluation, interviews were completed with 13 stakeholders. Data saturation was achieved.

All GPs interviewed are members of the CCG, unless identified as a 'university GP academic'. Those participants that are not board members (BM) are labelled as 'nonboard member (non-BM)' hereafter.

### Objective 1: to improve access to primary care by providing additional patient appointments

There was a demonstrable increase in GP appointments:

*'... the clinical bit seems to have worked out and access has improved.'* (GP CCG BM)

The actual number of appointments delivered, calculated by the CCG, was 18 266; 7.4% in excess of the target of 17 000 (unpublished data).

### Objective 2: to deliver service improvements through research and re-design

The CTFs provided clinical input into service development projects (such as urgent care, diabetes, and end of life care) within the CCG with mixed results in terms of their abilty to embed in these teams and influence projects.

### Objective 3: to raise the profile of Enfield for GPs to settle in the long term

Of the four postholders, two continued beyond the end of the post in Enfield practices and both have maintained a teaching commitment with UCL. The fifth directly-employed GP also continued to work in the area after the project. Two postholders did not complete the full duration of the programme; with one leaving to work abroad after 21 months, and the other due to family commitments after 11 months.

Undergraduate teaching was expanded in the locality and introduced into practices that were not previously involved. There were suggestions that this activity had an impact on the GPs, CTFs, and students in the practices in terms of their perceptions of Enfield as a place to work and study:

*'Proud to say that* [we] *have had medical students into the practice.'* (GP non-BM)

*'... more medical education will improve standards and the profile of the area.'* (GP non-BM)

*'When there is someone in the practice who is interested in teaching then it tends to ‘rub off’ on other colleagues' (GP non-BM).*

Action research, in terms of reflective practice and problem-solving as a community of practice, occurred throughout the project, but was particularly evident around issues such as practice allocation, CCG project development, and implementation of teaching in surgeries.

### Themes

From the qualitative data, four main themes emerged:

1. organisations working together;
2. recruiting GPs to underserved practices;
3. value for money; and
4. support and mentoring.

#### Organisations working together

Universities and PCOs are very different organisations with very different working methods and missions. A clear theme was the challenges for these organisations working together. This difficulty seemed to revolve around differing priorities within the shared project objectives, differing time scales, and differing cultures. The differing priorities revolved around service needs (delivering patient care and service redesign of clinical pathways for the PCO; teaching, CTF support, and — to a lesser extent — research for the university):

*'UCL’s main intention was teaching expansion and* [the] *CCG’s was increased appointment provision and clinical advice for their projects. Each stakeholder had its own objective and criteria of success for the project.'* (CTF)

*'*[The university] *had hoped for more junior research involvement by the CTF.'* (University manager)

The time scales and deadlines between institutions seemed to create difficulties. The funding for the project was for 2 years, but some of this time (but not funding) was eaten up at the start of the project, resolving issues such as how the CTFs should be employed and which practices should be allocated a CTF:

*'... there was a lot of bureaucracy with the university around recruiting to the post … I mean, NHS is a bureaucratic nightmare anyway ... but there was a 6–8 month delay in getting the adverts out and doing the recruitments. So that was an issue.'* (CCG manager)

Similarly, at the end of the project the CCG staff were worried about wider NHS reorganisation and the future of the CCG itself, meaning less time was focused on the CTF project:

*'As our financial situation has become more and more challenged, we were being told to shelve things that we were working on …'* (CCG manager)

*'We were able to secure some funding … which meant that we decided we would pilot some of the things from this financial year, but by the time we secured the funding and decided we were going to do that I heard that* [CTFs] *were leaving.'* (CCG manager)

The differing cultures of the organisations was seen in differing professional languages and time frames:

*'... although the primary care strategy came to an end on the 31st of March, this one project will run into the future, so it wasn’t completed by the end of the strategy period'.* (CCG manager)

Despite these difficulties, both sides seemed to place importance on developing the project and making the collaboration work:

*'I think we were both* [UCL and CCG] *working towards a common goal to give* [CTFs] *as good an experience as possible, we were both really keen and collaborated hard to do that.'* (CCG manager)

*'... it is vital to find a way of working together with CCGs, as it is important for a department of primary care to work with the local communities.'* (University manager)

#### Recruiting to underserved practices

The linkage of a university to the CCG post as a means of attracting recruits to the area was agreed to be successful in attracting high calibre GPs to work in the borough:

*'There was a good response when the CTF job was advertised ... These types of posts are vital as more interest by GPs is being shown for posts that contain a mixture of clinical practice and academia. This could be a solution to finding and retaining GPs.'* (University manager)

*'... bring value to Enfield in terms of the recruitment and hopefully retention of high calibre and more academic GPs, especially those newly qualified.'* (GP BM)

*'I think, from the point of view that four fantastic fellows have come to Enfield to work with our patients and introduce teaching in Enfield, then it has had value.'* (CCG manager)

One of the overarching aims of the project was attracting GPs to work in this underserved area and this was identified by participants:

*'Enfield needs more GPs ... to stay working here throughout their careers.'* (GP non-BM)

This aim was operationalised as getting the CTFs to work in small and developing practices. However, there were concerns about the suitability of many practices. These practices were often small practices with poor infrastructure:

*'... if the buildings are not fit for purpose you cannot have trainees* [CTFs] *in them.'* (GP non-BM)

They were also serving high demand communities ('Deep End' practices).^32^ These high clinical demands were identified by local GPs and CCG managers (regarding a host practice):

*'... it is ... a very tough practice, isn’t it?'* (CCG manager)

*'We have got a lot of deprivation and multiple ethnicities which make working as a GP challenging.'* (GP non-BM)

There was resistance from the university to placing relatively inexperienced GPs in struggling practices:

*'*[The university] *did not want the CTFs to be placed in unsuitable practices from a clinical point of view, i.e. places where they may have felt over-burdened. Hence the tension of … picking a small non-training/teaching practice which could overwhelm … the CTF.'* (University GP academic)

This transpired for at least one CTF:

*'... it was like a shot of cold water on my face. I really struggled in the first 3 months … The patient population is really, really challenging … I didn’t think I would survive but I have … and now I feel I am OK, because I was losing sleep, that is how bad it had gotten, but now I feel that I have found a way to work there.'* (CTF)

Many practices that might therefore benefit most from additional clinical capacity (effectively a free GP to the host practice) were, paradoxically, the least able to receive the support:

*'... non-training practices were meant to be selected but this idea got lost during first year, which was quite disappointing.* [It] *seemed to defeat the object.'* (GP CCG BM)

*'... we had to waive the issue of the* [non] *training practices because if we didn’t do that, then we would have no practices coming forward to be hosts.'* (CCG manager)

There seemed to be reticence of some of these practices to apply to be hosts; possibly because they were already struggling with workload, so didn’t have the resources to complete the application:

*'Probably it* [the application process] *is scary at first ... Unless you understand the system ... '* (Practice manager, non-BM)

*'... a lack of applications from practices with no training experience. Perhaps those practices felt overwhelmed.'* (University GP academic)

Additionally, some potential practices didn’t have enough consulting space to host another doctor or potential teaching space, which is the case for many inner city practices.^33^

Larger, more organised, better resourced practices ultimately were more successful in their applications, and their link to GPs who were board members of the CCG gave the perception of inequity, leading to some local unease:

*'... number of GPs … hadn’t realised that once they had stepped out of the steering group … they were conflicted, and wanted to actually apply to be hosts in their own right.'* (CCG manager)

*'... it* [the CTF programme] *was advertised for non-teaching practices. It was allocated to teaching practices.'* (GP non-BM)

This led to representation from the Local Medical Committee (LMC), the representative body for local NHS GPs,^34^ about the fairness of the process:

*'Although assurances were made regarding the objectivity of the selection process, the LMC continued to have concerns — this led to scepticism locally with scheme.'* (CCG manager)

#### Value for money

A recurring theme in the interviews and during the project was about the project’s value for money. Funding four full-time GPs was a considerable financial burden for a CCG. The university ceded its usual full economic costing to make it affordable, which meant the project’s finance was a source of concern for both organisations. However, several responders rated the project a success:

*'I can only talk about our practice … I feel that we benefitted.'* (GP non-BM)

*'... anecdotally, information from the practices about the positive effect within their practices also makes* [me] *feel it is a good investment.'* (GP BM)

*'... from the teaching point of view, the post has been successful.'* (University manager)

However, success was more often viewed through a lens of 'value for money' by key stakeholders:

*'... the post has not been value for money for UCL considering the time and personnel that has been required to run this scheme … The true cost for UCL was not covered.'* (University manager)

*'It will be ‘no’ in value for money.'* (CCG manager)

*'... hoping for a lot more from the post, but I feel that probably about 30% of these* [objectives] *have been achieved ... it is difficult to justify the funding allocated to it.'* (GP BM)

*'Is this something that CCGs want to hear i.e.* [only] *achieving subtle changes for large sums of money?'* (University GP academic)

In order to justfy the project to both organisations, the objectives that were set were overly ambitious and, in hindsight, unrealistic:

*'There were too many components to the post. Keeping the post realistic and not having over expectations of achievements from such a post is a learning point from this post.'* (CTF)

*'... there may have been too many aspects of this role which were too difficult to fulfil.'* (GP CCG BM)

So-called 'mission creep' was identified, as these objectives changed:

*'The expectations have changed of what has been expected of the CTF during the post. The role has varied.'* (University manager)

Despite the clear evidence that the project generated extra clinical capacity, some responders still felt the overall impact was minimal:

*'No, it is just a drop in the ocean … four fellows came … but how many will stay?'* (GP non-BM)

This raises the question whether this model of placing a CTF in a practice with multiple needs and no extra resource (beyond the additional clinical time that the CTF contributed, which was allocated to seeing patients) could ever make any changes. One responder felt the aim of

*'Increasing capacity … has been achieved. However, this could have been achieved simply by employing locums.'* (GP BM)

#### Support and mentoring

Due to the challenging nature of some of the practices and the wide scope of the project, the CTFs felt unsupported at times. The tight funding meant that there was little specific resource for mentoring and support:

*'I think ... we were hoping, relying on the clinical leads ... to provide that* [mentoring] *…* [They] *just couldn’t fit it in, I think, between* [their] *own clinical commitments and the other things we ask* [them] *to do.'* (CCG manager)

*'... there definitely should be a locally accountable clinician to guide the CTFs.'* (GP BM)

As a result of the lack of support from the CTFs' point of view, they migrated towards the university for support:

*Someone who could marry the CCG administrative parts and clinical side, and be a 'go to' person for us would have been helpful as we felt a little lost. We kept going back to UCL, as they are more used to mentoring.* (CTF)

*'... felt that the CCG lacked control over the project, as it was collaboration ... it’s been quite a messy arrangement for* [the CTFs] *as you had three masts, the CCG, UCL, and the practices.'* (CCG manager)

The fifth GP was appointed outside of the CTF collaboration towards the end of the project, perhaps to address some of the CCG’s concerns about control and direction of the project.

## Discussion

### Summary

This evaluation showed that universities can work with PCOs to promote recruitment of GPs in underserved areas. Using the brand or status of a university and making the role for the GP a varied one with service, teaching, and development can be attractive to GPs. However, several issues were identified that might be useful for those who may want to replicate such a scheme. Firstly, organisations need a stable collaboration to make such schemes work, with shared management and objectives from the outset. These objectives need to be clearly articulated and sufficiently robust that they can weather changing circumstances in both organisations. Unless very experienced practitioners (with consequent higher salary and project costs) are being employed, then there needs to be adequate mentorship (separate from line management).^35^ There needs to be time to allow new appointees to settle into a post and to develop professional relationships before they can begin to initiate any kind of service change. Finally, any objectives set need to be limited in scope and realistic; if a GP is providing clinical service to an inner city practice, that is highly likely to spill over beyond any notional session time and will have an impact on their ability to undertake wider developmental roles. The consequences of these factors — lengthy posts, sustained support, and limited objectives — mean that they will be a significant financial burden on small PCO or similar organisation. That the collaboration unravelled and diverged towards the end of the project perhaps attests to the difficulty in sustaining such collaborations longer-term. However, the benefits of this model beyond patient contact are important: teaching medical students in practice is known to have an impact on career choice,^36^ and on the choice of their working location;^37–39^ teaching within a practice has a positive impact on practitioners^40^ and their patients;^41^ and there is value in developing links between PCOs and university departments of general practice.^21,42^

Each of these attributes could probably be achieved more cheaply by employing locums to see more patients, incentivising GPs to work in an area,^43^ incentivising practices to teach or train, or the direct funding of researchers by CCGs,^29^ but narrow-focused models may not have the additive effect of this model.

### Strengths and limitations

This approach enabled a low-cost means of evaluating a project that might otherwise go unevaluated, and generated useful data. The evaluation also captured a cross-disciplinary range of stakeholders’ views.

There was no specific funding for this evaluation, so the researchers were the object of the evaluation. This will very likely have had an impact on what participants chose to disclose. While measures were taken to minimise this risk with interview schedules, there was evidence of participants who were known to be unhappy with the project not participating. Additionally, some participants were extremely cautious (they were perceived by the researchers as being 'very diplomatic') in what they would discuss. The summarised interviews may also have lost some of their context without the verbatim quotes.

### Comparison with existing literature

The identified literature highlights many similar themes to this project. Specifically in relation to longer-term recruitment, the different models varied in their success. In the Durham model, it is suggested that 10–40% of postholders stay on in the locality;^5^ 75% of the LATS trainees stayed on London,^15^ although this may just reflect a desire to stay in the capital. Retention in the south Wales scheme was high, at 61%.^9^ More broadly, there were recognised benefits to health authorities, academic departments of general practice, and GPs working together.^15,20^ Specifically, the CeMENT project reported evidence of the positive effects on GP from undergraduate teaching.^33^ However, there is surprisingly little published evidence of the impact of these models on GP recruitment in inner city areas.^37^

At an organisational level, there are difficulties with any health reorganisation,^18,23^ and difficulties in establishing collaboration due to organisational tensions.^44^ Within these collaborations, decisions and strategies will change over time, and will move away from their initial objectives,^13^ but short term financial imperatives (usually cost savings) will usually win out over quality improvement activities.^42^ This has been described as 'conflicting aims; interface issues; organizational problems; and leadership tensions'.^44^

At the level of the specific interaction between PCOs and academic GP departments, the tensions identified here are echoed in other studies.^6^ Specifically for this project, the long lead-time or bureaucratic delays reported here have been observed elsewhere.^15^ One evaluation captures the governance issue of employment of GPs by institutions (such as universities) that are not providing GP services.^9^

At the level of the individual GPs (be they fellows, LATS, or inner city lecturers), there is a clear need for mentoring during these projects,^5^ which is usually provided by academic departments.^9,45^ Practice-based mentoring caused issues in this study, but has been successful elsewhere.^5^ However, peer support groups of these GPs appears to be an important element for project success.^5,9,45^ This occurred spontaneously, locally, with this project but was not formalised. The ability to deliver local service development projects by fellows is highlighted, balanced against the very real issue of multiple competing priorities.^9,45^ The need for continuity for the CTF in a single practice has been identified as necessary in order for them to become part of the practice team,^5^ but in this project this was sacrificed to ensure equality of distribution of GPs to the practices.

For the host practices, the ability to apply and access such projects poses difficulties,^14^ but this can be mitigated by a 'whole locality' approach.^15^ Space — particularly in small, single-handed or inner city practices — often acts as a structural barrier to project participation.^33^ For individual GPs within these practices or PCOs, there are often challenges with hosting, such as mentoring junior GPs,^5^ engaging with practice changes, and the impact on the clinical service.^9^ There are often financial conflicts of interest (around receiving free GPs) which need to be acknowledged and managed in a transparent manner.^46^

Financial instability and unsustainability of these projects is widely reported,^6,16^ with only the Welsh model, which had high level government support, seemingly able to provide a long-term mechanism to ensure survival.^9^

### Implications for research

This study shows that an academic institution can collaborate with a PCO to assist in recruitment of GPs to underserved areas, although there remain questions about financial sustainability. If mechanisms could be found to fund clinical time via the normal GP General Medical Services contract, this would substantially reduce project costs. The fact that these models have reappeared over the last 20–30 years suggest, while they are of interest to PCOs, they may not be financially or managerially sustainable.
